# Supplementary material for: Antagonism of interferon signaling by fibroblast growth factors promotes viral replication
Source: EMBO Mol Med. 2020 Jul 27;12(9):e11793. doi: 10.15252/emmm.201911793 (PMC7507082; doi:10.15252/emmm.201911793)
Supplement: Supplementary file 1 — Appendix [file EMMM-12-e11793-s001.pdf]

Supporting Information

Maddaluno, Urwyler, Rauschendorfer et al.

Antagonism of interferon signaling by fibroblast growth factors promotes viral replication

Table of contents:

Appendix Fig S1: Verification of the efficiency of different inhibitors of FGFR signaling proteins

Appendix Fig. S2: Proteasome inhibition abolishes the effect of FGF7 on ISG expression

Appendix Table S1: Antibodies used for Western blot and/or immunostaining

Appendix Table S2: Primers used for qRT-PCR

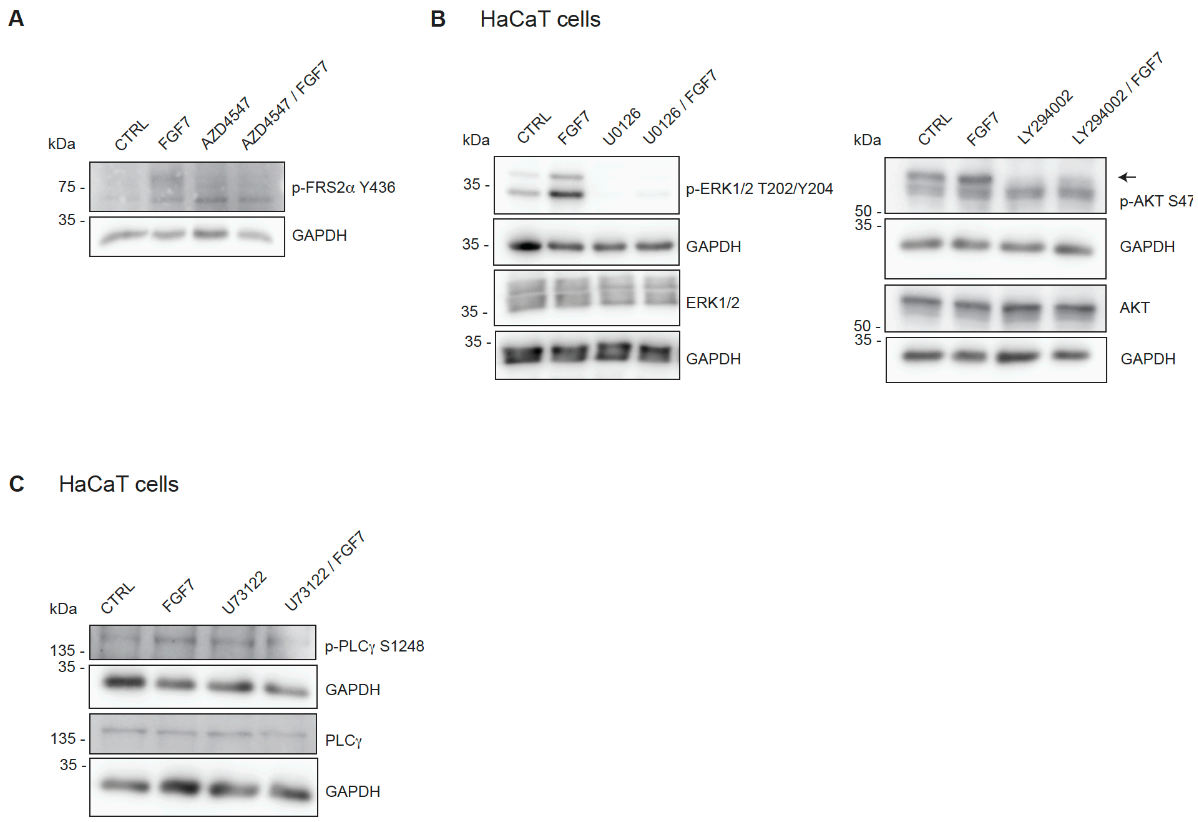

Appendix Fig S1 Verification of the efficiency of different inhibitors of FGFR signaling proteins

(**A-C**) Serum-starved HaCaT keratinocytes were pre-treated with the FGFR inhibitor AZD4547 (1 µm) (A), the MEK1/MEK2 inhibitor UO126 (10 µM) (B, left panel), the PI3K inhibitor LY294002 (5 µM) (B, right panel), or the PLC-γ inhibitor U73122 (5 µM) (C) or vehicle and then incubated with FGF7 for 15 min. Cell lysates were analyzed by Western blot for total and phosphorylated FRS2α (A), pERK1/2 and AKT (B), pPLC-γ (C) and GAPDH (loading control).

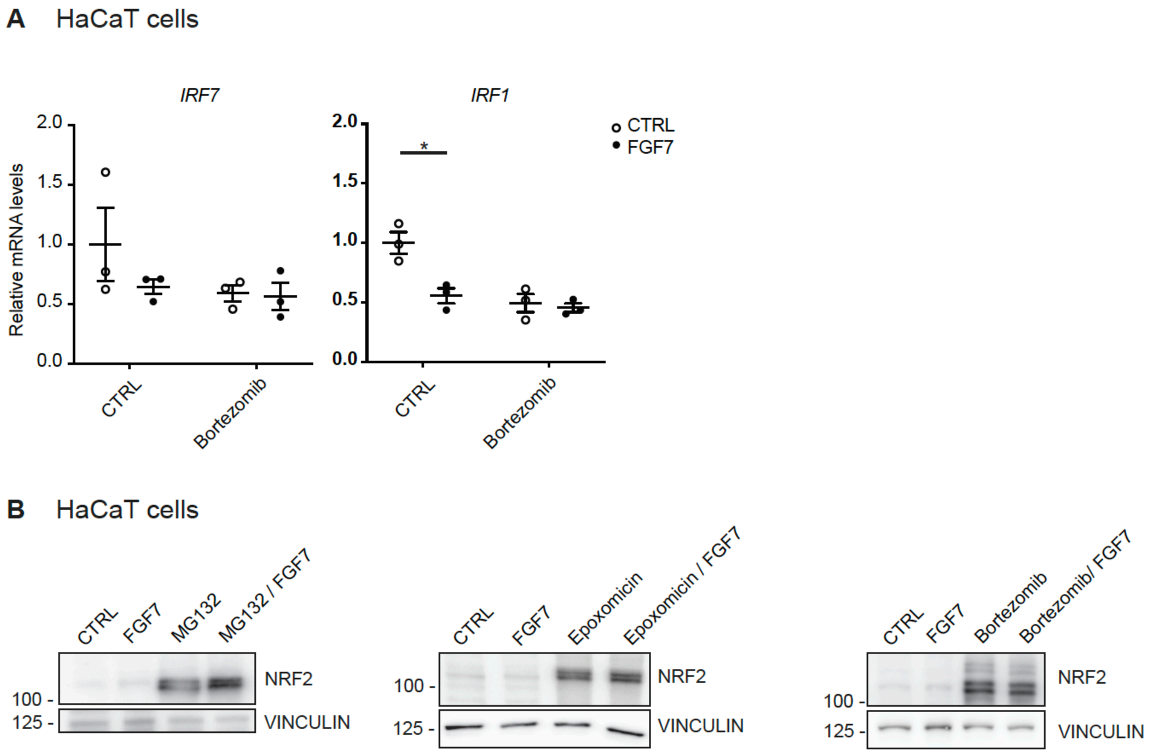

Appendix Fig. S2 Proteasome inhibition abolishes the effect of FGF7 on ISG expression

(**A**) Serum-starved HaCaT keratinocytes were pre-treated for 2 h with the proteasome inhibitor bortezomib (10 μm), followed by a 6 h treatment with FGF7 (10 ng/ml) or vehicle. RNA samples were analyzed by qRT-PCR for *IRF7* and *IRF1* relative to *RPLP0*.

(B) To test the activity of the proteasome inhibitors used for the experiments shown in Fig. 2G, H and Appendix Fig. S2A, HaCaT keratinocytes were treated as described in the legends of these figures and analyzed by Western blot for expression of NRF2 and vinculin (loading control).

Data information: Scatter plots show mean +/- S.E.M. Mean expression levels in CTRL cell cultures was set to 1, and mRNA levels relative to this value are shown. N=3 per treatment group from one experiment. ***$P \leq 0.001$ (t test with Welsh correction).

Appendix Table S1: Antibodies used for Western blot and/or immunostaining

Antigen	Cat. No.	Dilution	Company/Source
IRF1	8478	1:1000	Cell Signaling, Danvers, MA
IRF3	11904	1:1000	Cell Signaling
Phospho-IRF3	4947	1:1000	Cell Signaling
IRF7	Sc-9083	1:400	Santa Cruz, Santa Cruz, CA
IRF7	4920	1:1000	Cell Signaling
RSAD2	13996	1:1000	Cell Signaling
IRF9	76684	1:1000	Cell Signaling
Lamin A/C	4777	1:2000	Cell Signaling
Phospho-STAT1 (Y701)	9167	1:1000	Cell Signaling
Phospho-STAT1 (S727)	8826	1:1000	Cell Signaling
STAT1	610115	1:500	BD Biosciences, San José, CA
RSAD2	13996	1:1000	Cell Signaling
Phospho-STAT2 (Y690)	88410	1:1000	Cell Signaling
STAT2	4594	1:1000	Cell Signaling
GAPDH	5G4	1:10000	HyTest, Turku, Finland
Zona Occludens 1 (ZO.1)	339100	1:500	Invitrogen, Carlsbad, CA
Keratin 14 (K14)	PRB-155P	1:1000	Biolegend, San Diego, CA

Vinculin	v4505	1:2000	Sigma, Munich Germany
Flavivirus group antigen antibody (clone D1-4G2-4-15)	MAB10216	1:400	Merck-Millipore, Darmstadt, Germany
Herpes Simplex Virus 1 (HSV-1) glycoprotein D (Glyc-D)	ab27586 and ab6507	1:1000	Abcam, Cambridge, UK
LCMV nucleoprotein (clone VL-4)		1:400	Kindly provided by Prof. Rolf Zinkernagel, Zurich, Switzerland
AF555-conjugated anti-mouse IgG	A-21422	1:400	Thermo Fisher, Waltham, MA
AF555-conjugated anti-rabbit IgG	A-21428	1:400	Thermo Fisher
AF488-conjugated anti-mouse IgG	A-11001	1:400	Thermo Fisher

Appendix Table S2: Primers used for qRT-PCR

Primers used for mouse samples:

Target gene	Primers (forward and reverse)
Ifit1	5`-AGC AAC CAT GGG AGA GAA TGC-3`; 5`-CT TTC AGG TGC CTC ACG TA-3
Irf7	5`-AGC TTG GAT CTA CTG TGC GC-3`; 5`-GGG TTC CTC GTA AAC ACG GT-3`
Oasl2	5`-TGC CTG GGA GAG AAT CGA AG-3`; 5`-AGC CTC CCT TCA CCA CCT TA-3`
Rps29	5`-GGT CAC CAG CAG CTC TAC TG-3`; 5`-GTC CAA CTT AAT GAA GCC TAT GTC C-3`

Stat1	5`-GGA TCG CTT GCC CAA CTC T-3`; 5`-GCA GAG CTG AAA CGA CCT AGA-3`
Rsad2	5`-GGA GGT GGT GCA GGG ATT AC-3`; 5`-GGA AAA CCT TCC AGC GCA CA-3`
Stat2	5`-CTT TTG CAA GCG AGA GAG CC-3`; 5`-TGA AGC GCA GTA GGA AGG TG-3`
Ifnb	5`-CTG GCT TCC ATC ATG AAC AA-3`; 5` -CAT TTC CGA ATG TTC GTC CT-3`
Ifnl3	5`-GGT TGG AGG TGA CAG AGT-3`; 5`-AAG GGT GCC ATC GAG AAG-3`
Fgf7	5`-CCT TTG ATT GCC ACA ATT CC-3`; 5`-CAA ACG GCT ACG AGT GTG AA-3`
Cre	5`-CGA CCA GGT TCG TTC ACT CA-3`; 5`-CGA GTT GAT AGC TGG CTG GT-3`

Primers used for human samples:

**Target gene**	**Primers (forward and reverse)**
DUSP6	5`-GTT CTA CCT GGA AGG TGG CT-3`; 5`-AGT CCG TTG CAC TAT TGG GG-3`
IFIT1	5`-AGC TTA CAC CAT TGG CTG CT-3`; 5`-CCA TTT GTA CTC ATG GTT GCT GT-3`
IRF1	5`-CTC TGA AGC TAC AAC AGA TGA G-3`; 5`-GTA GAC TCA GCC CAA TAT CCC-3`
IRF3	5`-TCG TGA TGG TCA AGG TTG T-3`; 5`-AGG TCC ACA GTA TTC TCC AG-3`
IRF7	5`-AGC TGT GCT GGC GAG AAG-3`; 5`-CTC TCC AGG AGC CTT GGT TG-3`
OAS2	5`-GGG CTA TTT CCA GAC AAC GC-3`; 5`-GAA AAC CAG GCC TGT GAT CTT GG-3` 463
ISG15	5`-ACT CAT CTT TGC CAG TAC AGG AG-3`; 5`-CAG CAT CTT CAC CGT CAG GTC-3`

NECT1	5`-CTA CCA CAT GGA CCG CTT CAA G-3`; 5`-CTT TGC AGG TGA GCT TCA CGT C-3`
RPLP0	5`-CCA CAT TGT CTG CTC CCA CA-3`; 5`-GAA GAC AGG GCG ACC TGG AA-3`
RSAD2	5`-GCT GCT AGC TAC CAA GAG GAG-3`; 5`-ATC TTC TCC ATA CCA GCT TCC-3`
STAT1	5`-AAA GGA AGC ACC AGA GCC AAT-3`; 5`-TCC GAG ACA CCT CGT CAA AC-3`
STAT2	5`-GGA TCC TAC CCA GTT GGC TG-3`; 5`-GAG GGT GTC TTC CCT TTG GC-3`
OAS1	5`-TTC CTC CCT GCC ATT CAT CC-3`; 5`-TCC AGA AAC CCT CGA TTG TGA-3`
MxA	5`-ACC TAC AGC TGG CTC CTG AA-3`; 5`-GCA CTC AAG TCG TCA GTC CA-3`
DDX58	5`-ATC CCA GTG TAT GAA CAG CAG-3`; 5`-GCC TGT AAC TCT ATA CCC ATG TC-3`
CGAS	5`-GGG AGC CCT GCT GTA ACA CTT CTT AT-3`; 5`-CCT TTG CAT GCT TGG GTA CAA GGT-3`
TLR3	5`-TGG TTG GGC CAC CTA GAA GTA-3`; 5`-TCT CCA TTC CTG GCC TGT G-3`
IFNB	5`-TGG GAG GAT TCT GCA TTA CC -3`; 5`-CAG CAT CTG CTG GTT GAA GA -3`
IFNL1	5`-GGT GAC TTT GGT GCT AGG-3`; 5`-TGA GTG ACT CTT CCA AGG-3`
RPL27	5`-AAA GCT GTC ATC GTG AAG AAC-3`; 5`-GCT GCT ACT TTG CGG GGG TAG-3`
SOCS1	5`-GGA ACT GCT TTT TCG CCC TTA -3`; 5`-AGC AGC TCG AAG AGG CAG TC-3`
SOCS3	5`-GGC CAC TCT TCA GCA TCT C-3`; 5`-ATC GTA CTG GTC CAG GAA CTC-3`
