# Supplementary material for: Antagonism of interferon signaling by fibroblast growth factors promotes viral replication
Source: EMBO Mol Med. 2020 Jul 27;12(9):e11793. doi: 10.15252/emmm.201911793 (PMC7507082; doi:10.15252/emmm.201911793)
Supplement: Supplementary file 2 — Expanded View Figures PDF [file EMMM-12-e11793-s002.pdf]

Expanded View Figures

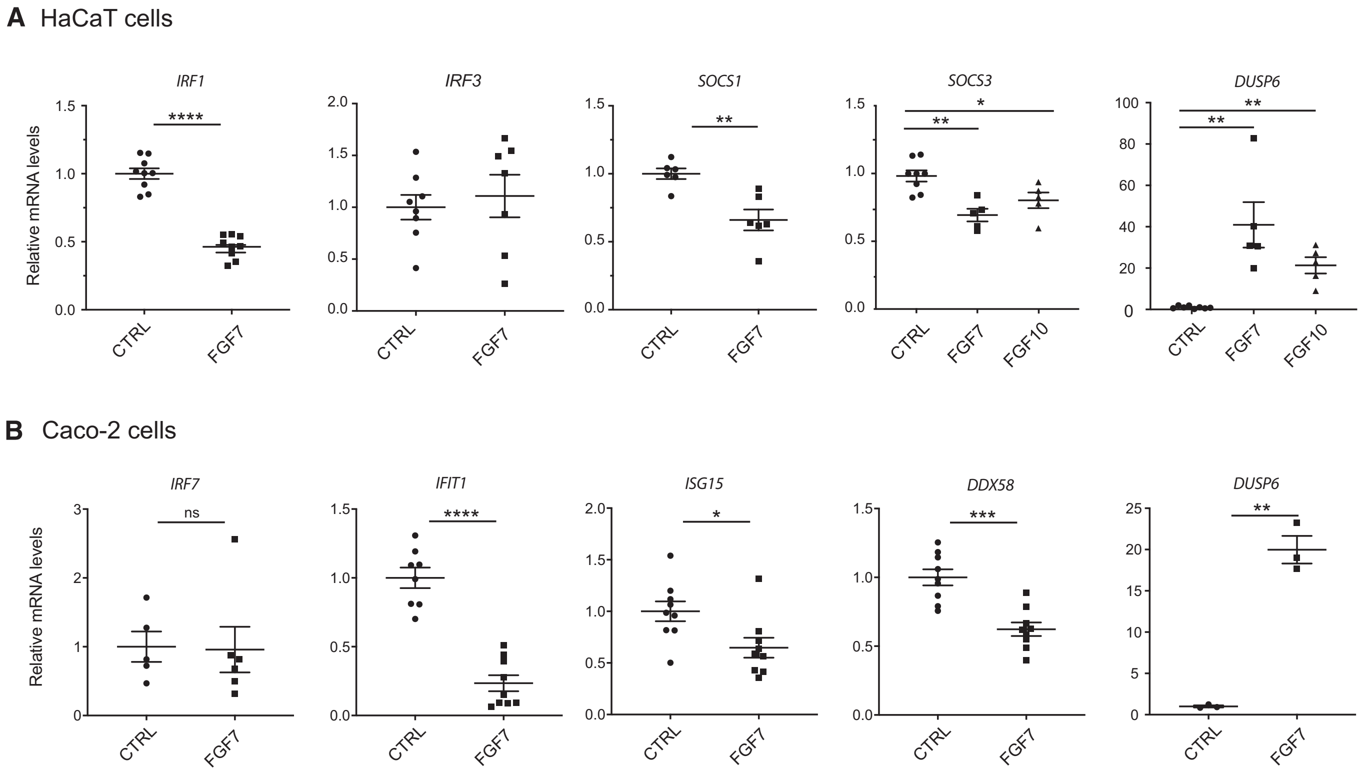

Figure EV1. FGF signaling suppresses ISG expression in HaCaT keratinocytes and Caco-2 colon cancer cells.

A Serum-starved HaCaT keratinocytes were treated for 16 h with FGF7 or FGF10 (10 ng/ml each). RNA samples were analyzed by qRT–PCR for *IRF1, IRF3, SOCS1, SOCS3, and DUSP6* relative to *RPLP0*.

B Serum-starved Caco-2 cells were treated for 6 h with FGF7 (10 ng/ml). RNA samples were analyzed by qRT–PCR for *IRF7*, *IFIT1*, *ISG15*, *DDX58*, or *DUSP6* relative to *RPLP0*.

Data information: Scatter plots show mean ± SEM. Mean expression levels in CTRL cell cultures were set to 1. (A) N = 5–9 from 2 to 3 experiments, (B) N = 5–8 from 3 experiments for ISGs, N = 3 from 1 experiment for *DUSP6*. ns: non-significant, *$P \leq 0.05$, **$P \leq 0.01$, ***$P \leq 0.001$, ****$P < 0.0001$ (Mann–Whitney U-test). Exact P-values are provided in Dataset EV2.

 © 2020 The Authors

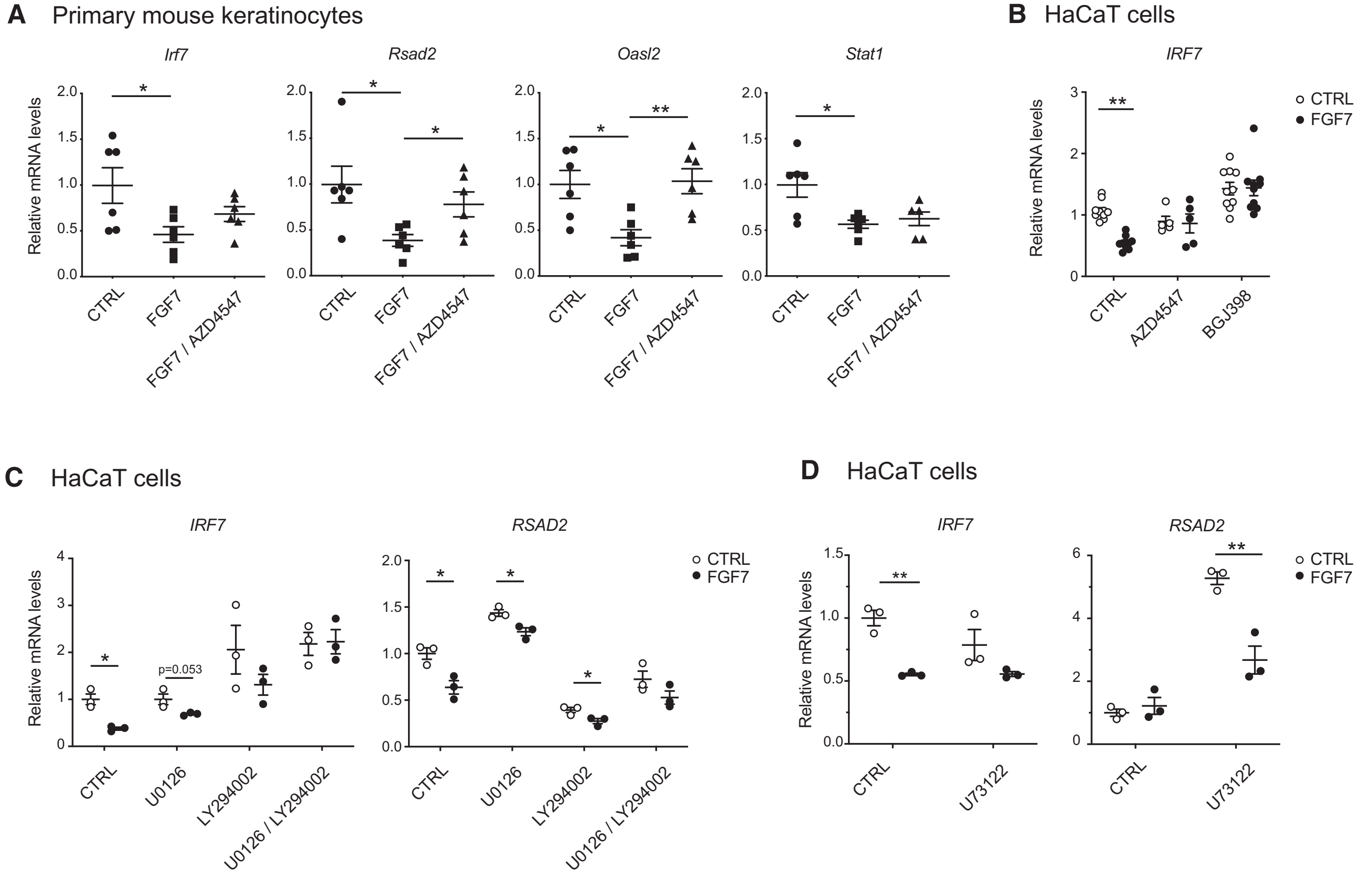

Figure EV2. FGF7 suppresses ISG expression in keratinocytes via FGFR-mediated MEK1/2-ERK1/2 and PI3K-AKT signaling.

A Growth factor-starved primary mouse keratinocytes were treated for 6 h with FGF7 (10 ng/ml) in the presence of absence of the FGFR kinase inhibitor AZD4547 (1 μM) (added 3 h prior to FGF7). RNA samples were analyzed by qRT–PCR for expression of *Irf7*, *Rsad2*, *Oasl2*, and *Stat1* relative to *Rps29*.

B Serum-starved HaCaT cells were treated for 6 h with FGF7 (10 ng/ml) in the absence or presence of the FGFR kinase inhibitors AZD4547 (1 μM) or BGJ398 (3.5 μM) (added 2 h prior to FGF7) and analyzed by qRT–PCR for *IRF7* relative to *RPLP0*.

C, D Serum-starved HaCaT keratinocytes were treated for 6 h with FGF7 (10 ng/ml) in the absence or presence of the PI3K inhibitor LY294002 (5 μM) and/or the MEK1/2 inhibitor U0126 (10 μM) (C) or the PLC-γ inhibitor U73122 (5 μM) (D) (added 2 h prior to FGF7) and analyzed by qRT–PCR for *IRF7* and *RSAD2* relative to *RPLP0*.

Data information: Scatter plots show mean ± SEM. Mean expression levels in CTRL cell cultures were set to 1. (A) $N = 5$–6 from 2 experiments, (B) $N = 5$–10 from 4 experiments. (C, D) Representative experiments from at least two independent experiments are shown, $N = 3$ per experiment. *$P \leq 0.05$, **$P \leq 0.01$ (Mann–Whitney *U*-test (A, B) and *t*-test with Welch correction for assessment of FGF7 effect (C, D). Exact *P*-values are provided in Dataset EV2.

© 2020 The Authors

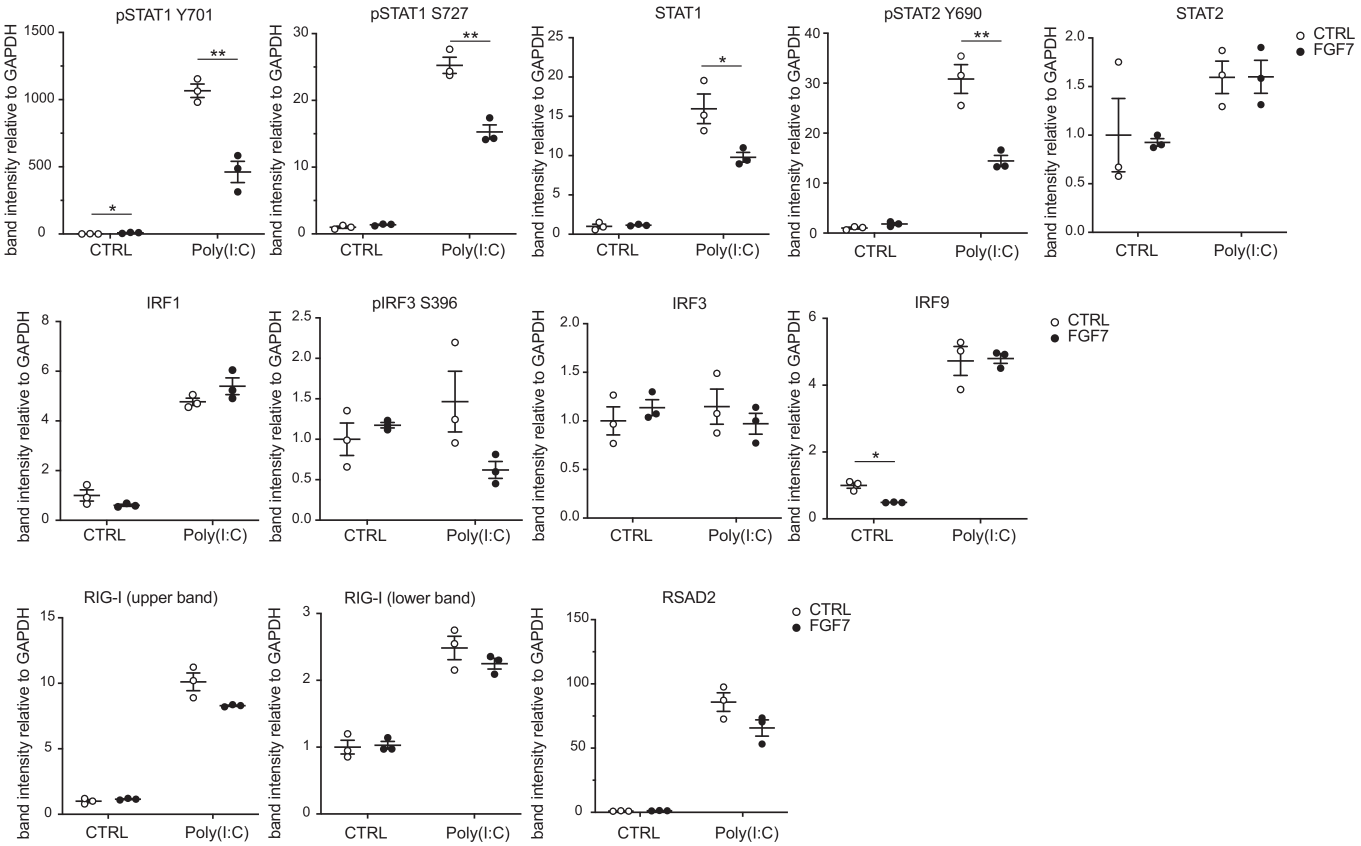

Figure EV3. FGF7 suppresses ISG expression and poly(I:C)-induced STAT1 and STAT2 activation.

Quantification of the Western blot data shown in Fig 4B. Band intensities (based on densitometry) were normalized to the intensities of the GAPDH bands. Scatter plots show mean ± SEM ($N = 3$). Mean band intensity in the CTRL cell cultures was set to 1. *$P \leq 0.05$, **$P \leq 0.01$ (t-test with Welch correction for assessment of FGF7 effect). Exact P-values are provided in Dataset EV2.

 © 2020 The Authors
