# Supplementary figures and images for: Antagonism of interferon signaling by fibroblast growth factors promotes viral replication
Source: EMBO Mol Med. 2020 Jul 27;12(9):e11793. doi: 10.15252/emmm.201911793 (PMC7507082; doi:10.15252/emmm.201911793)

**Figure 1G**

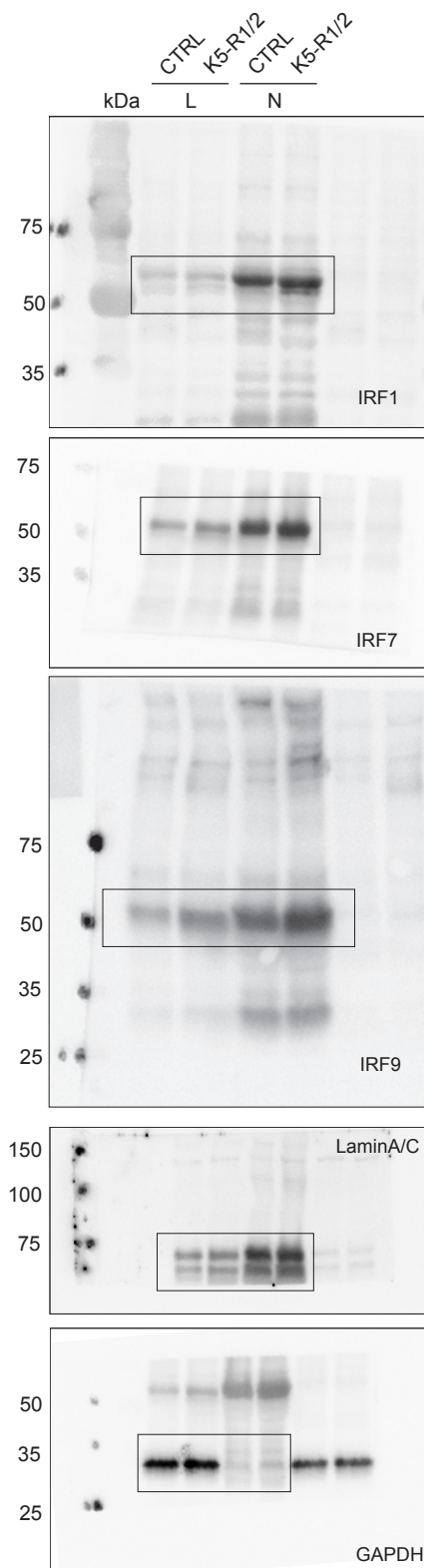

Supplement: Supplementary file 6 — Source Data for Figure 1 [file EMMM-12-e11793-s005.pdf]

FIGURE 2C

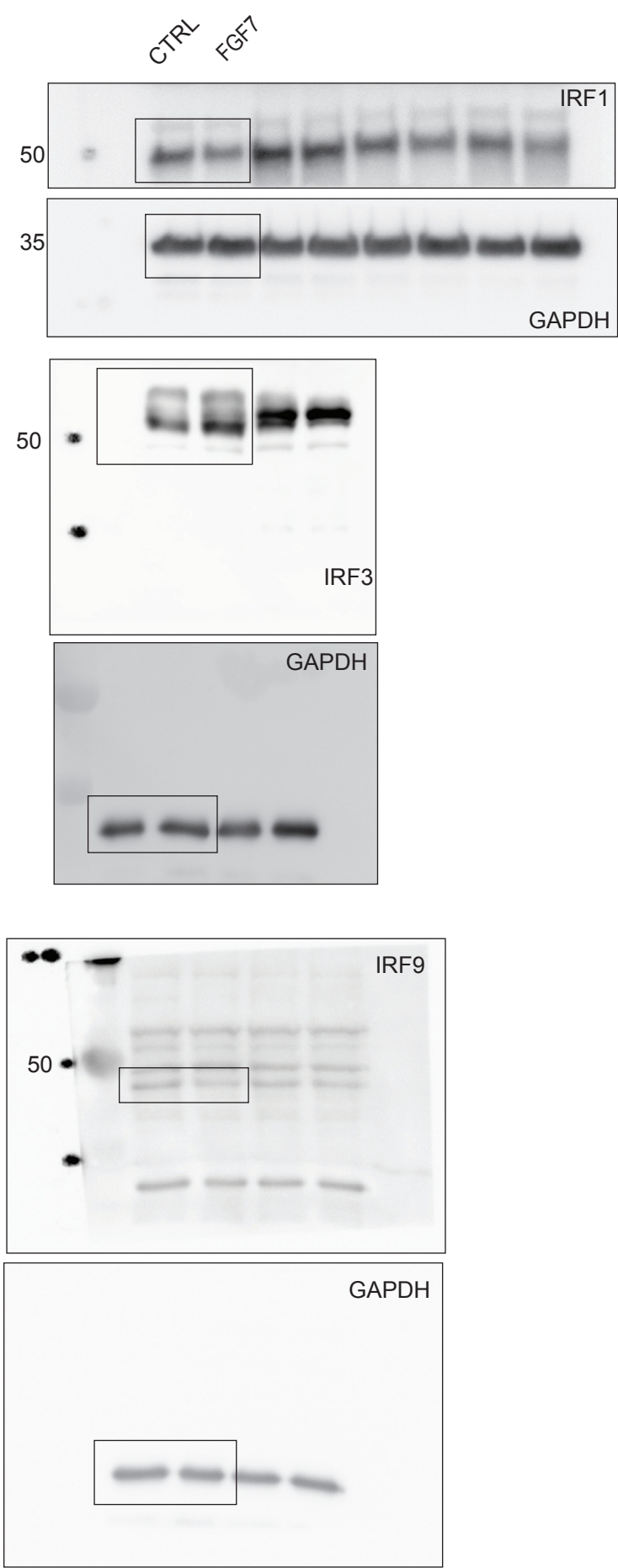

Supplement: Supplementary file 7 — Source Data for Figure 2 [file EMMM-12-e11793-s006.pdf]

FIGURE 3C

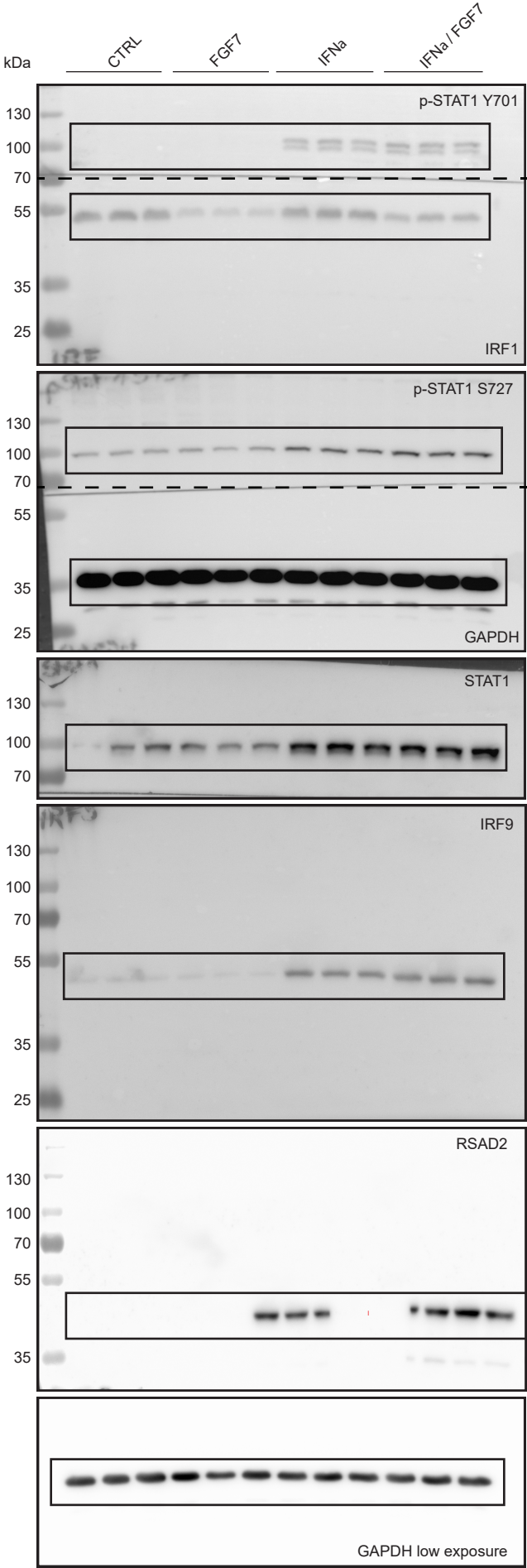

Supplement: Supplementary file 8 — Source Data for Figure 3 [file EMMM-12-e11793-s007.pdf]

FIGURE 4B

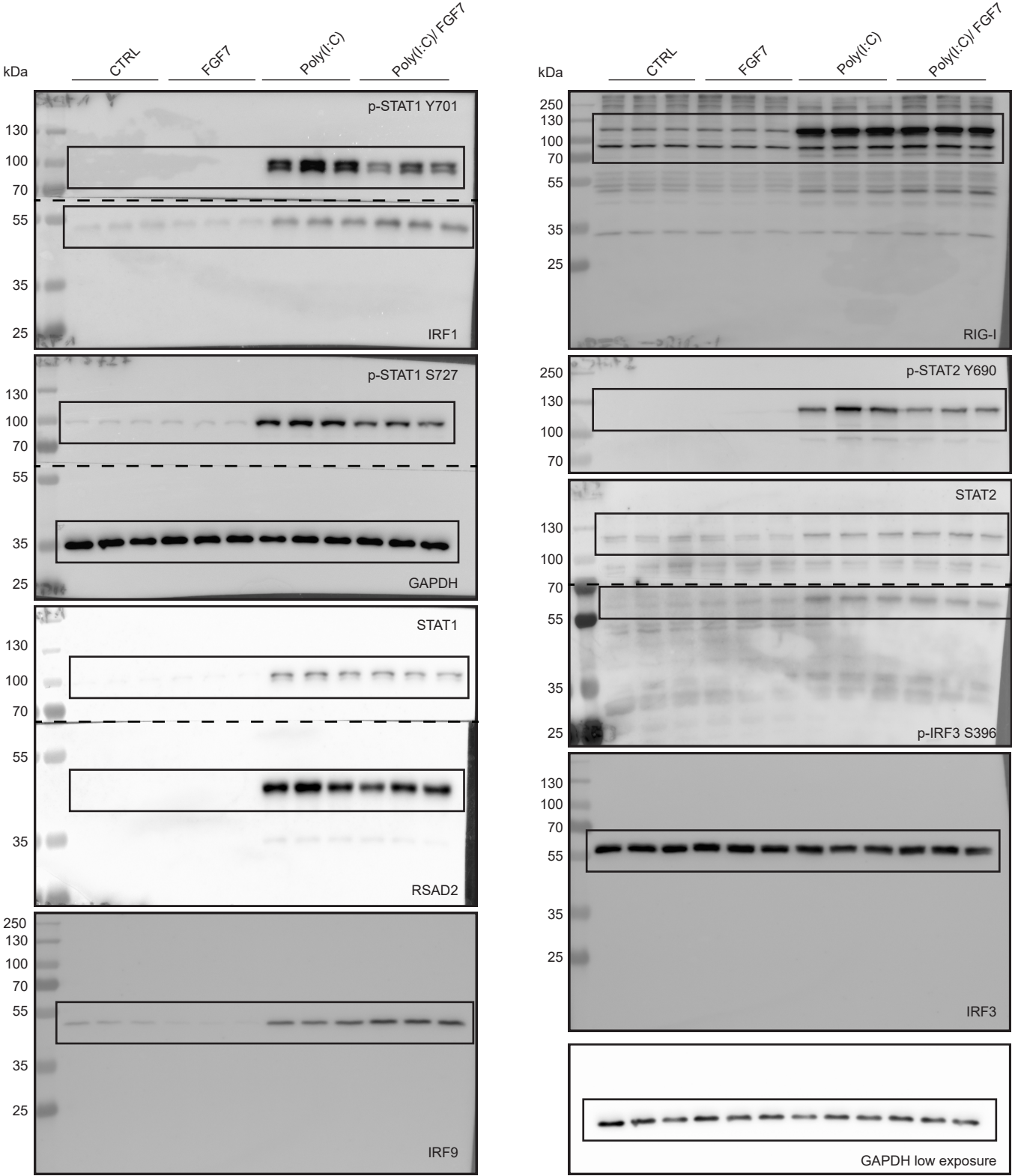

Supplement: Supplementary file 9 — Source Data for Figure 4 [file EMMM-12-e11793-s008.pdf]

**Figure 5**

**F**

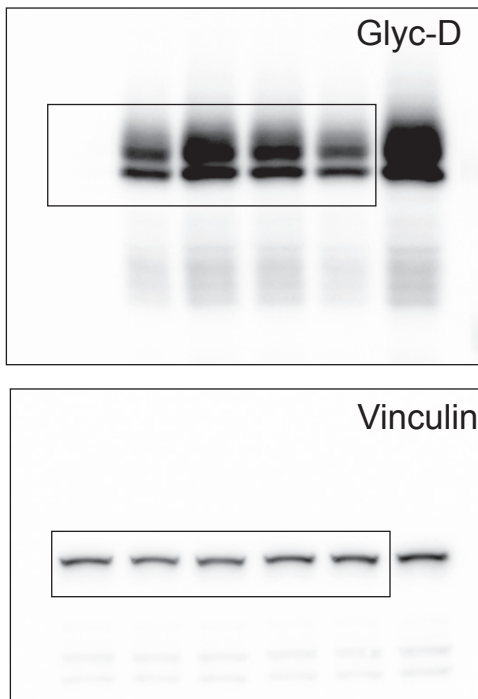

**H**

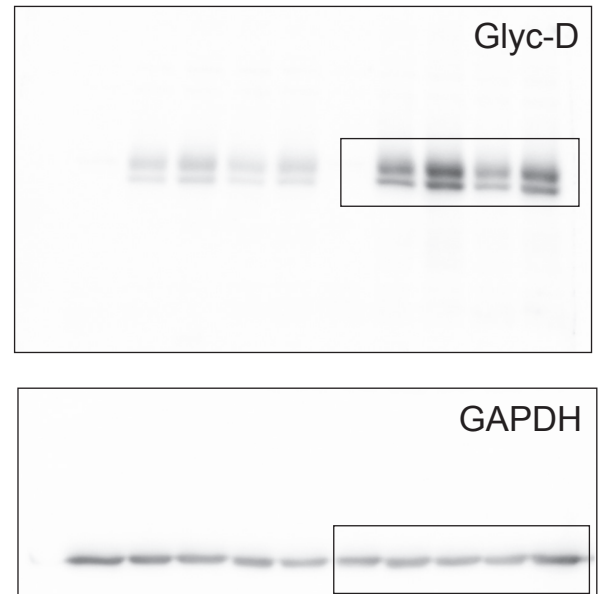

Supplement: Supplementary file 10 — Source Data for Figure 5 [file EMMM-12-e11793-s009.pdf]

FIGURE 6E

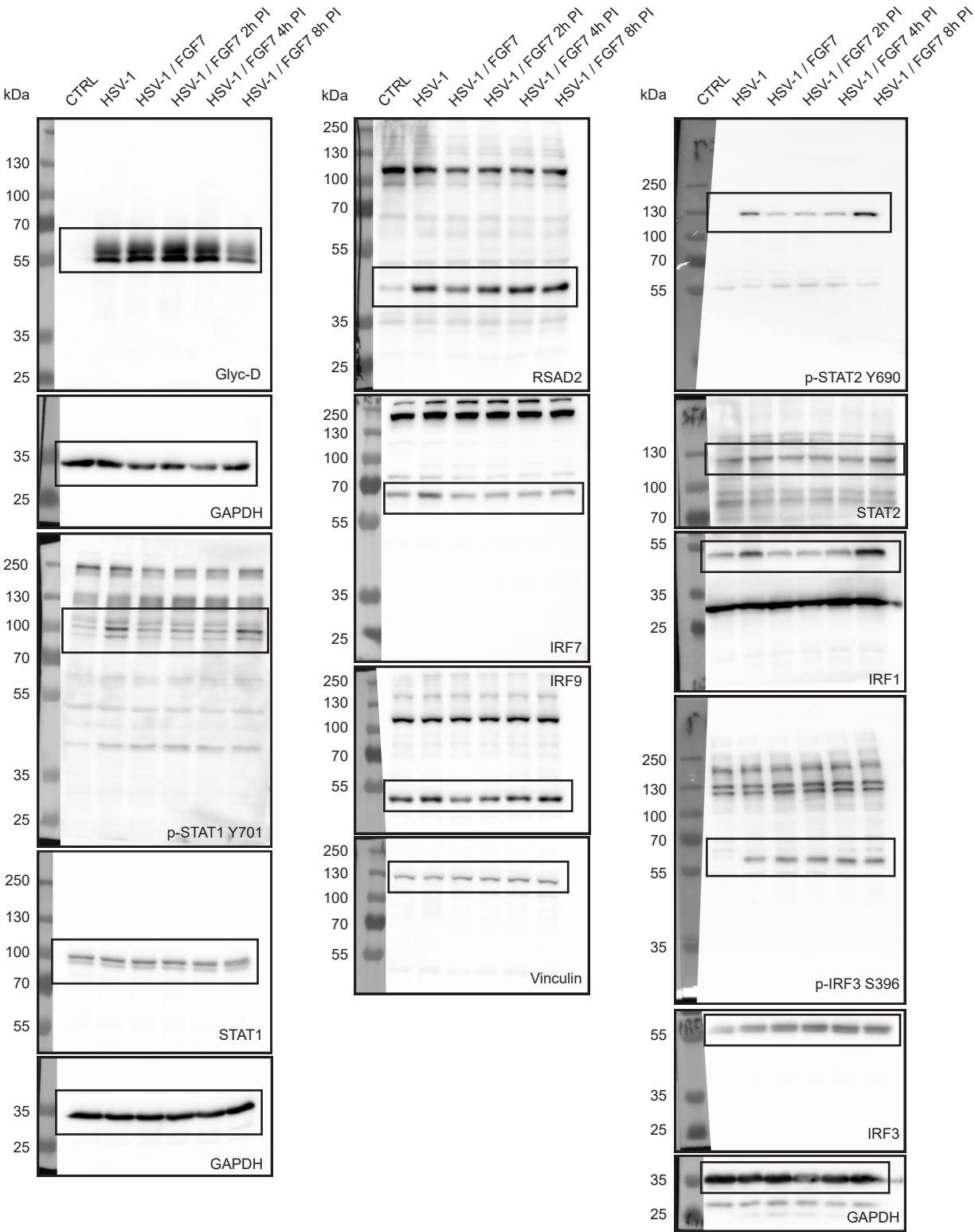

Supplement: Supplementary file 11 — Source Data for Figure 6 [file EMMM-12-e11793-s010.pdf]

Figure 7A

H

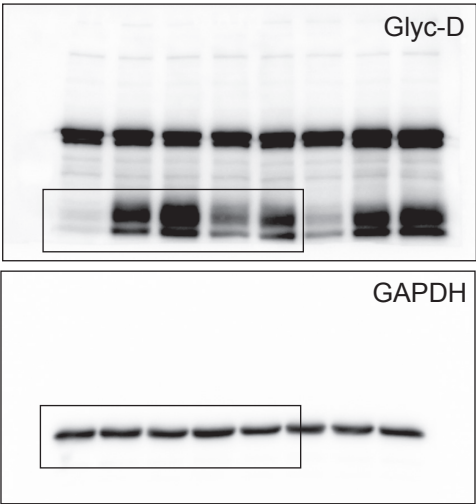

Supplement: Supplementary file 12 — Source Data for Figure 7 [file EMMM-12-e11793-s011.pdf]
